# New insights into root 2-naphtholic acid exudation, microbiome recruitment, and designing of drought-smart future crops

**DOI:** 10.3389/fpls.2026.1938013

**Published:** 2026-10-16

**Authors:** Md Atikur Rahman, S. M. Shahinul Islam, Nadiyah M. Alabdallah, Salman Latif, Mahmudul Hoque, Md. Mahadi Hasan

**Affiliations:** 1 Plant Biotechnology and Genetic Engineering Lab, Institute of Biological Sciences, University of Rajshahi, Rajshahi, Bangladesh; 2 Department of Biology, College of Science, Imam Abdulrahman Bin Faisal University, Dammam, Saudi Arabia; 3 Department of Chemistry, College of Science and Humanities in Al-Kharj, Prince Sattam bin Abdulaziz University, Al-Kharj, Saudi Arabia; 4 Performance Measurement Unit, Vice Presidency for Scientific Research & Innovation, Imam Abdulrahman Bin Faisal University, Dammam, Saudi Arabia; 5 Basic and Applied Scientific Research Center, Imam Abdulrahman Bin Faisal University, Dammam, Saudi Arabia

**Keywords:** 2-naphtholic acid, drought tolerance, root exudate, soil microbiome, soybean

## 1 Introduction

Soybean (*Glycine max* L.), which is the world’s most economically important legume and an excellent source of plant-based protein and oils, is acutely vulnerable to drought (Touma et al., 2015). The grain-filling stage in soybean is highly sensitive to drought stress, which can reduce yield by approximately 40%–70% (Zhang et al., 2022). Conventional breeding for drought tolerance is slow and resource-intensive, and irrigation capacity is becoming increasingly constrained. Microbiome-based interventions, therefore, have attracted attention as a complementary, comparatively low-input route to resilience. This type of intervention operates operation nutrient metabolism, osmotic adjustment, phytohormone modulation, and microbiome signaling (de Vries et al., 2020; Gahlot, 2026).

The rhizosphere is where this change takes place. Root exudates, including organic acids, sugars, amino acids, and secondary metabolites, serve simultaneously as a carbon source for microbial catabolism and as chemical signals that structure community assembly (Zhalnina et al., 2018; Fan et al., 2025). Stressed plants are widely believed to modify exudation in order to enlist beneficial taxa, the “Cry-for-help” model, although this hypothesis has recently been criticized for resting on correlative evidence (Liu et al., 2024; Tharp et al., 2026). A largely unexplored corollary is the reverse direction of causality: whether the diversity of the resident soil community itself determines which chemical signals a plant is able to deploy.

Chen et al. (2026) address precisely this question. By combining dilution-to-extinction across three orders of magnitude of soil microbial diversity with metabolomics, transcriptomics, bacterial genetics, and biophysical binding assays, the authors show that high rhizosphere diversity enables the drought-specific accumulation of 2-naphthoic acid (2-NA). This root exudate recruits the keystone rhizobacterium Sinorhizobium CS204 and reprograms its nitrogen (N) metabolism.

In this paper, we offer perspectives on the topic and outline new directions for future research. First, microbial diversity should be treated as an upstream constraint on host chemical signaling rather than as a correlate of plant performance. Second, 2-NA is best read as a proof of principle for multifunctional exudates, not as a candidate agricultural input. Third, the distance between the mechanism and field deployment is considerably greater than current enthusiasm for microbiome engineering suggests.

## 2 Microbial diversity as an upstream constraint on exudate chemistry

The novel insights from Chen et al. (2026) demonstrate that drought-stressed plants grown within the highest-diversity community (D1) accumulated 2-NA at approximately 16.5 μg·mL^-^¹, while intermediate diversity (D2) yielded around 0.27 μg·mL^-^¹, and the lowest diversity soil (D3) yielded none. Because 2-NA was undetectable in well-watered plants, drought appears to act as the trigger and diversity as the gain control. Integrated transcriptomic and metabolomic profiling linked 2-NA accumulation to phenylpropanoid-related metabolism, which is consistent with broader evidence that community context reshapes plant secondary metabolism under abiotic stress (Li et al., 2025; Kazarina et al., 2025; Yang et al., 2025).

Our reading inverts conventional framing. The “cry-for-help” narrative casts the plant as the agent and microbiome as the respondent, suggesting the resident community sets the boundaries for what the plant can do. Low-diversity rhizospheres especially showed far broader transcriptional reprogramming than high-diversity ones. We interpret this as a metabolic economy: where a diversity community supplies functional insurance, the host can invest narrowly and precisely; where it does not, the plant is pushed towards costly, diffuse, and less targeted responses (Yang et al., 2021; Domeignoz-Horta et al., 2024). On this view, diversity is chemical signaling capital.

This carries an uncomfortable agronomic implication. Intensive tillage, monoculture, and heavy agrochemical loading erode rhizosphere diversity (Xiang et al., 2025). If it is correct that drought-adaptive signaling depends on a diverse resident community, then biodiversity loss may progressively disable the very recruitment mechanisms that microbiome-based agriculture seeks to harness. An important caveat should temper this interpretation. Dilution-to-extinction alters richness but does not hold composition constant, because rare taxa and their functional genes are lost together. “Diversity” is therefore an operational rather than a clean variable here, and the design cannot separate the effect of species richness itself from the loss of specific keystone lineages. Resolving this will require synthetic communities of matched composition but varied richness, assembled across contrasting soils.

## 3 2-naphthoic acid as a tri-functional mediator: what is new and what is not yet shown

Exudate-mediated recruitment is not itself novel: malic acid attracts Bacillus subtilis, strigolactones recruit arbuscular mycorrhizal fungi, and flavonoids initiate rhizobial nodulation through NodD (Ruan et al., 2026; Qiao et al., 2024). What distinguishes 2-NA is that a single benzoid appears to play three roles at once (Chen et al., 2026; Niu et al., 2025). It acts as a chemoattractant sensed by three methyl-accepting chemotaxis protein (ΔmcpU, ΔmcpY, and ΔmcpZ), with a deletion mutant showing substantially reduced chemotaxis and root colonization. It serves as a nutritional reward taken up through an ATP-binding cassette (ABC) transporter system encoded with *upgBAEI*. And it functions as a regulatory ligand that binds N-cycling protein NirK, NapA, and FixI, confirmed by molecular docking and microscale thermophoresis, with *napA* expression strongly induced in co-culture. Comparable metabolite-driven shaping of rhizosphere bacteria has been reported during drought and rehydration in other systems (Lei et al., 2025; Zhao et al., 2025).

We regard this tri-functionality as the paper’s most transferable idea, and we would expect analogous multifunctional metabolites to be discoverable in other crops. These limitations deserve emphasis. The biosynthetic route remains inferred rather than demonstrated, the phenylpropanoid association is correlative, and no 2-NA biosynthetic gene has been identified or knocked out in planta. The regulatory mechanism rests substantially on *in vitro* binding and docking, which establishes physical interaction but not *in situ* flux control. Finally, chemoattraction was assayed against a limited panel of strains, so the specificity of 2-NA for beneficial taxa as opposed to the many soil bacteria that metabolize aromatic compound remains open. Until these gaps are closed, 2-Na is a compelling mechanistic exemplar rather than an established lever.

## 4 Nitrogen-cycle reprogramming: benefits and an emissions caveat

The functional payoff reported by Chen et al. (2026) is nutritional. Under drought, restricted mass flow limits N delivery, impairing protein synthesis, photosynthesis and reactive-oxygen scavengers (de Vries et al., 2020). Co-application of 2-NA with *Sinorhizobium CS204* increased plant biomass by roughly 38%, improved photosynthesis and stomatal performance, altered the expression of antioxidant and drought-responsive genes, and raised tissue concentration of several mineral nutrients (**Figure 1**). This is consistent with growing evidence that rhizobia influence soil N cycling well beyond classical nodule symbiosis and a field demonstration proved that improved rhizobial inoculants can raise nodulation, N- fixation, and legume yield (Ling et al., 2022; Shah et al., 2024).

**Figure 1:**
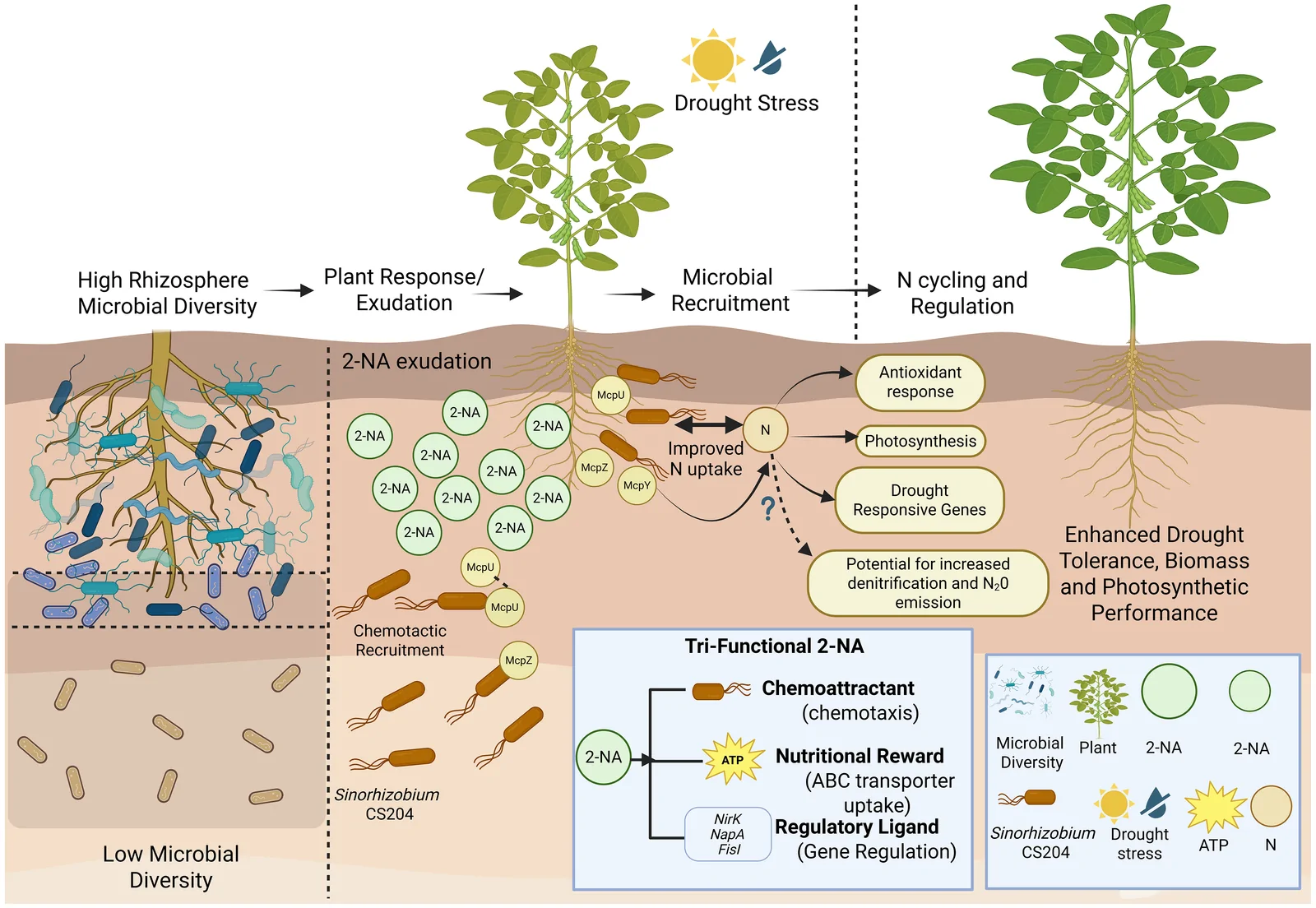
Conceptual model of diversity-driven 2-NA signaling and drought tolerance in soybean plants. Under drought conditions, high rhizosphere microbial diversity leads to the drought-specific accumulation of 2-NA in root exudates. This process helps chemotactic recruitment and colonization of *Sinorhizobium* CS204. It also enables 2-NA mediated modulation of bacterial N-cycling and improves N-acquisition, photosynthetic performance, biomass yield, and drought tolerance in soybean. Arrows indicate the direction of the signal or material flux. 2-NA, 2-naptholic acid; N, nitrogen.

One consequence deserves more scrutiny than it has received. The treatment reportedly upregulates denitrification (NirK, NapA, NorB, and NosZ) alongside N-fixation. Denitrification is not a uniformly benign process: incomplete denitrification is a major source of nitrous-oxide, a greenhouse gas with high radiative forcing, and drying-rewetting cycles are known hotspots for such emissions. A drought-mitigation strategy stimulates denitrification and could therefore trade water-stress resilience against climate burden. No N_2_O flax data accompany the current work and, in our view, no scald application should be contemplated until the NosZ-to-NirK balance and the resulting N_2_O:N_2_ product ratio are measured directly in soil.

## 5 From control systems to the field

The gap between these findings and agricultural practice is substantial, and it should be stated plainly rather than bridged rhetorically. Persistence is the first obstacle: naphthalene-derived acids are readily biodegraded from common soil bacteria and absorb strongly to organic matter and clays, so the effective rhizosphere concentration of an applied dose is unlikely to resemble that achieved in controlled systems. Soil type, pH, temperature, and moisture regime will all modulate this, and more has been varied systematically.

Second, ecological selectivity is unproven at community scale. Applying an aromatic acid to soil may preferentially favor aromatic-degrading taxa, some of which are opportunists, and could shift community structure in ways that are unhelpful or difficult to reverse. Third, the biosafety and regulatory position of applying a naphthalene derivative to agricultural soil is not trivial and has not been addressed. Fourth, the economics of synthesizing and delivering a defined metabolite at field scale, alongside a live inoculant with its own formulation and shelf-life constraints, remain unexplored.

These considerations argue for two parallel and non-exclusive strategies. The more immediately actionable is restoration of soil microbial diversity through reduced tillages, cover cropping, diversified rotation, and organic amendment, supporting the endogenous capacity for 2-NA-mediated recruitment. The more speculative is targeted co-application of a metabolite with a compatible keystone strain as a biostimulant, which should be treated as research program rather than a near-term product. Progress of both will depend on standardized multi-omics frameworks capable of linking microbial community composition to function across sites and seasons, an area where methodological harmonization remains a recognized bottleneck (Kumar et al., 2023).

## 6 Concluding remarks and prospects

Chen et al. (2026) provide the most comprehensive mechanistic framework to date for how resident microbial diversity conditions a plant’s chemical recruitment of beneficial partners under drought. We see this as evidence that diversity is a functional prerequisite for practice host signaling and that individual exudates can be multifunctional in ways that classical signal-versus-substrate distinctions do not capture.

The priorities we would set are correspondingly modest and testable: identification and genetic validation of the 2-NA biosynthesis pathway; measurement of exudation kinetics under realistic, fluctuating drought rather than imposed step changes; determination of the fate, half-life, and sorption behavior of 2-NA across contrasting soils; assessment of community-level and N_2_O consequences; and examination of whether diversity-conditioned signaling operates in cereals and other legumes (Gahlot, 2026). Multi-side field evaluation should follow, not precede, these steps. Framed this way, the contribution of this work is conceptual rather than immediately applied. It does not yet deliver drought-smart agriculture but clarifies what such a strategy would need to be built upon and suggests that protecting the diversity already present in agricultural soils may be a more reliable investment than engineering it. However, this study opens new dimensions of research on the diversity-dependent chemical ecology of drought adaptation in soybean that incorporates plant metabolomics, bacterial sensor biology, and N biogeochemistry, which merely resolves a long-standing gap in our understanding of the diversity function relationship in the rhizosphere and microbiome-assisted climate-smart crop improvement.
